# Estimating Zika virus attack rates and risk of Zika virus-associated neurological complications in Colombian capital cities with a Bayesian model

**DOI:** 10.1098/rsos.220491

**Published:** 2022-11-30

**Authors:** Kelly Charniga, Zulma M. Cucunubá, Diana M. Walteros, Marcela Mercado, Franklyn Prieto, Martha Ospina, Pierre Nouvellet, Christl A. Donnelly

**Affiliations:** ^1^ Medical Research Council Centre for Global Infectious Disease Analysis, Department of Infectious Disease Epidemiology, Imperial College London, London, UK; ^2^ Instituto Nacional de Salud, Bogotá, Colombia; ^3^ School of Life Sciences, University of Sussex, Brighton, UK; ^4^ Department of Statistics, University of Oxford, Oxford, UK

**Keywords:** arboviruses, emerging infectious diseases, reporting rates, outbreak analysis, Guillain–Barré syndrome, mathematical modelling

## Abstract

Zika virus (ZIKV) is a mosquito-borne pathogen that caused a major epidemic in the Americas in 2015–2017. Although the majority of ZIKV infections are asymptomatic, the virus has been associated with congenital birth defects and neurological complications (NC) in adults. We combined multiple data sources to improve estimates of ZIKV infection attack rates (IARs), reporting rates of Zika virus disease (ZVD) and the risk of ZIKV-associated NC for 28 capital cities in Colombia. ZVD surveillance data were combined with post-epidemic seroprevalence data and a dataset on ZIKV-associated NC in a Bayesian hierarchical model. We found substantial heterogeneity in ZIKV IARs across cities. The overall estimated ZIKV IAR across the 28 cities was 0.38 (95% CrI: 0.17–0.92). The estimated ZVD reporting rate was 0.013 (95% CrI: 0.004–0.024), and 0.51 (95% CrI: 0.17–0.92) cases of ZIKV-associated NC were estimated to be reported per 10 000 ZIKV infections. When we assumed the same ZIKV IAR across sex or age group, we found important spatial heterogeneities in ZVD reporting rates and the risk of being reported as a ZVD case with NC. Our results highlight how additional data sources can be used to overcome biases in surveillance data and estimate key epidemiological parameters.

## 1. Introduction

Zika virus disease (ZVD) is an emerging infectious disease caused by Zika virus (ZIKV), a flavivirus that is most commonly spread by *Aedes aegypti* mosquitoes in tropical regions [1]. Although most people infected with ZIKV experience mild or no symptoms, the virus has been associated with an increased risk of congenital birth defects and neurological complications (NC) in adults, including Guillain–Barré syndrome (GBS), encephalitis, myelitis and meningoencephalitis [2–9].

GBS symptoms consist of tingling, numbness or pain in the limbs as well as limb weakness and hypo- or areflexia [10]. The majority of patients with this condition require hospitalization with some needing intensive care and ventilatory support [11]. About 3–10% of GBS patients die [12]. Although most patients fully recover, some may experience long-term morbidity, including depression and disability [13,14]. Acute GBS can be treated with intravenous immunoglobulin and plasma exchange [14].

The baseline worldwide incidence of GBS has been estimated at 1.1–1.8 cases per 100 000 population per year [15]. Research suggests that the risk of GBS tends to be higher for males than females and increases with age [16]. The onset of neurological symptoms is often preceded by a viral or bacterial infection, especially *Campylobacter jejuni* [17]. However, the epidemiology of GBS in Latin America and the Caribbean is not well understood [18].

ZIKV caused a major epidemic in the Americas between 2015 and 2017, and Colombia was one of the most affected countries. From August 2015 to June 2017, Colombia reported 106 033 suspected and confirmed ZVD cases (Instituto Nacional de Salud, INS, data). By early 2018, 248 cases of confirmed congenital syndrome associated with ZIKV infection had been reported to the Pan American Health Organization (PAHO) [19]. In addition, 418 cases of NC among suspected or confirmed ZVD cases had been identified (INS data).

Previous studies on the ZIKV epidemic in Colombia have estimated epidemiological parameters, including ZIKV infection attack rates (IARs) and reporting rates of ZVD as well as the risks of congenital birth defects and NC in adults [20–26]. The IAR is the proportion of the at-risk population that is infected over a specific period. A related quantity is the reporting rate, or the proportion of infections that are ultimately reported as cases of the disease. Both are of considerable interest during and after an epidemic. Surveillance systems do not capture all cases which leads to uncertainty in the ‘true’ incidence of disease. Cases can be missed due to (i) under-ascertainment of infections at the community level and (ii) underreporting of infections at the healthcare level [27]. As surveillance data are used to guide resource allocation and estimate epidemiological parameters, it is important to understand how accurately they reflect the true burden of disease in populations and adjust observed incidence with multiplication factors as needed. Accurate estimates of IARs are critical for understanding population-level susceptibility and anticipating the timing of future epidemics.

Modelling studies, community-based studies and serological surveys have all been used to estimate IARs, reporting rates and the risk of NC for the ZIKV epidemic in Colombia. The community-based studies and the serological surveys focused on a small number of cities [20,28], while the modelling studies have largely concentrated on national and sub-national (department) levels [21–23,26]. Although estimates at large spatial scales can be useful, the risk of ZIKV transmission varies across Colombia due to vast differences in elevation and climate which impact mosquito biology. Thus, more estimates at smaller spatial scales are needed to improve inference about epidemic dynamics and minimize bias [29]. Moreover, ZIKV IARs, reporting rates of ZVD and the risk of NC are difficult quantities to estimate for a variety of reasons, including the low rate of symptomatic disease and co-circulation with other arboviruses; both factors impact data quality. Therefore, it is important to estimate these quantities using different datasets, which may capture different aspects of the underlying disease transmission patterns, and methods to optimize planning and resource allocation.

The aim of this research is to estimate ZIKV IARs, reporting rates of ZVD and the risk of NC following ZIKV infection in departmental capital cities of Colombia. Although all cities in this analysis are considered at-risk for arbovirus transmission, we hypothesized that there would be spatial heterogeneity in the estimates due to differing geography and climate. We also set out to assess how the parameters vary by age and sex. Women of child-bearing age were prioritized for ZIKV surveillance during the epidemics, but the extent to which the surveillance data were biased toward this demographic had not previously been quantified.

## 2. Methods

### 2.1. Data

We analysed anonymized line list data on suspected and laboratory-confirmed cases of ZVD reported to Sivigila, Colombia's national public health surveillance system, between 2015 and 2017. We also analysed a dataset on patients with NC and recent febrile illness compatible with ZVD as well as seroprevalence data. The analysis was restricted to capital cities at risk of arbovirus transmission because seroprevalence data were only available for four capital cities (Cúcuta, Neiva, Medellín and Sincelejo) [28]. Colombia is organized into 32 departments (administrative level 1) and 1122 municipalities (or cities, administrative level 2) [30]. Each department has a capital city, and Bogotá is the capital of both the country and the department of Cundinamarca. The location used in this study corresponds to the location of likely infection, which was determined by the clinician who reported the case. The location of likely infection is preferred over residence because it accounts for travel to areas with higher risk of arbovirus transmission.

**Figure 1.**
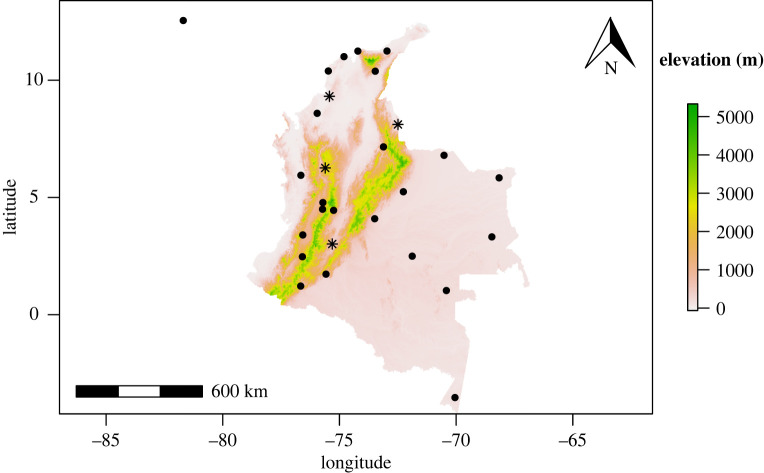
Locations and elevations of the 28 capital cities. Stars indicate the four cities with seroprevalence data, while points indicate the 24 cities without seroprevalence data. To create the map, SRTM 90 m Digital Elevation Data were downloaded from the CGIAR-CSI GeoPortal [31]. The data were cropped using an administrative level 0 shapefile for Colombia obtained from the Humanitarian Data Exchange [32]. The shapefiles were prepared by OCHA and are available under a CC BY-IGO licence [33]. No changes were made to the shapefiles.

Out of 32 capital cities, 28 were considered at-risk, reporting 54 737 ZVD cases and 212 cases of ZIKV-associated NC (table 1 and figure 1). We calculated Pearson's correlation coefficient for this relationship. The populations of the 28 cities considered comprise 28% of Colombia's total population of about 49 million. See electronic supplementary material for a full description of the data.

**Table 1.** Epidemiological and demographic data for 28 Colombian capital cities. NC, neurological complications; ZIKV, Zika virus; ZVD, Zika virus disease.

city	department	population in 2016	reported cases of ZIKV-associated NC	reported suspected and laboratory-confirmed cases of ZVD	estimated post-epidemic seroprevalence (95% CI)
Arauca	Arauca	89 712	1	788	
Armenia	Quindío	298 199	0	189	
Barranquilla	Atlántico	1 223 616	80	4665	
Bucaramanga	Santander	528 269	8	4322	
Cali	Valle del Cauca	2 394 925	23	16 279	
Cartagena	Bolívar	1 013 389	4	1021	
Cúcuta	Norte de Santander	656 380	44	6485	0.479 (0.440–0.519)
Florencia	Caquetá	175 407	3	663	
Ibagué	Tolima	558 805	3	4076	
Inírida	Guainía	19 983	0	12	
Leticia	Amazonas	41 639	0	278	
Medellín	Antioquia	2 486 723	8	549	0.067 (0.048–0.090)
Mitú	Vaupés	31 861	0	17	
Mocoa	Putumayo	42 882	1	57	
Montería	Córdoba	447 668	4	1785	
Neiva	Huila	344 026	13	3409	0.578 (0.538–0.618)
Pereira	Risaralda	472 000	0	463	
Popayán	Cauca	280 054	0	51	
Puerto Carreño	Vichada	16 000	0	17	
Quibdó	Chocó	115 907	0	14	
Riohacha	La Guajira	268 712	0	279	
San Andrés	San Andrés and Providencia	71 946	0	1109	
San José del Guaviare	Guaviare	65 611	0	154	
Santa Marta	Magdalena	491 535	2	1913	
Sincelejo	Sucre	279 031	6	856	0.659 (0.620–0.696)
Valledupar	Cesar	463 219	2	788	
Villavicencio	Meta	495 227	5	2377	
Yopal	Casanare	142 979	5	2121	

### 2.2. Bayesian hierarchical model

**Figure 2.**
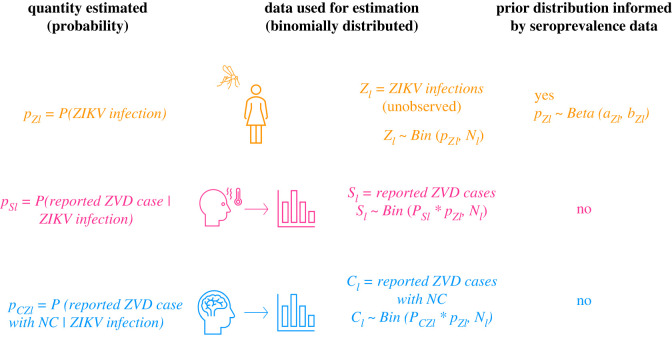
Schematic diagram of Bayesian hierarchical model framework. Estimated probabilities are shown in relation to the data used for estimation and model equations where *l* is location. The probability of ZIKV infection is shown in the first row in orange. The probability of reporting a case of ZVD per ZIKV infection is shown in the second row in pink, and the probability of reporting a case of ZVD with NC is shown in the third row in blue.

We used a Bayesian hierarchical model to estimate the following probabilities from observed data via a binomial sampling process: (i) the probability of ZIKV infection *p_Zl_*, or IAR, (ii) the probability of reporting a case of ZVD per ZIKV infection *p_Sl_*, or reporting rate and (iii) the probability of reporting a case of ZVD with NC *p_CZl_*, or reporting rate of ZIKV-associated NC, where *l* denotes location (city) [21]. The probabilities of interest were estimated simultaneously for the four cities with seroprevalence data as well as 24 other capital cities (figure 2). Overall (non-location specific) estimates of the probabilities were also generated. The total number of ZIKV infections *Z_l_* and the number of infections that go on to be reported as either suspected or laboratory-confirmed cases *S_l_* in each city *l* are binomially distributed as

$$Z_{l}∼\mathrm{Bin}(\;p_{Zl},N_{l}),$$

and

$$S_{l}∼\mathrm{Bin}(\;p_{Sl}*p_{Zl},N_{l}),$$

where *N_l_* is the population size of each city. For city *l* with seroprevalence data, the prior distribution for *p_Zl_* was

$$p_{Zl}∼\mathrm{Beta}(a_{Zl},b_{Zl}),$$

where the method of moments was used to determine *a_Zl_* and *b_Zl_*. Means and variances corresponding to a uniform random variable with the possible range of IARs were specified using the optim function in R. The range of IARs was defined by the 95% confidence interval of the post-epidemic seroprevalence. In this way, the seroprevalence data informed the prior distributions while still permitting values outside the observed ranges. For cities without serological data and the overall probability of ZIKV infection (*p_Z_*), *a_Zl_* and *b_Zl_* were both set to 1 which is equivalent to a uniform distribution between 0 and 1.

The risk of developing NC following ZIKV infection was assumed to be similar across cities; however, reporting rates were expected to differ. The model accounted for these differences by assigning hyperprior distributions to *p_Sl_* and *p_CZl_* which were used to estimate the overall risk of being reported as a ZVD case (*p_S_*) and a ZVD case with NC (*p_CZ_*), respectively.

$$S∼\mathrm{Bin}(\;p_{S}*p_{Z},N)$$

and

$$C∼\mathrm{Bin}(\;p_{CZ}*p_{Z},N),$$

where *C* is the total number of ZIKV-associated NC that were reported during the epidemic. Finally, the following equation estimated the total number of NC due to ZIKV infection in city *l*:

$$C_{l}∼\mathrm{Bin}(\;p_{CZl}*p_{Zl},\;N_{l}).$$

There were 91 total parameters in the model (87 excluding the hyperprior distributions).

After performing the analysis on all data from the 28 cities, we classified the data by sex and age group (electronic supplementary material). Age was dichotomized into 0–39 years and greater than or equal to 40 years (the median age of patients with ZIKV-associated NC was 41 years, and the NC data were too sparse to split into more than two age groups). Although the seroprevalence estimates for Cúcuta, Neiva, Medellín and Sincelejo were sex-specific, Nouvellet *et al*. (P.N., personal communication, 2021) found no statistically significant differences between males and females. Consequently, we fitted the model to both sexes simultaneously assuming the same IAR for each sex. We fitted the model by age group in the same way. Each of these models had 153 parameters. Further details on the modelling framework can be found in the electronic supplementary material.

### 2.3. Expected number of excess NC reported per 10 000 reported cases of ZVD

We estimated the number of ZIKV-associated NC per 10 000 reported ZVD cases for each city and overall. To obtain the estimate for each city, we merged four Markov chain Monte Carlo chains of *p_CZl_* and *p_Sl_* after removing the burn-in. Then, we divided the merged samples of *p_CZl_* by those of *p_Sl_* for each city and multiplied the results by 10 000. To obtain the overall estimate, we pooled the samples of *p_CZl_* and *p_Sl_* across all cities before the division step.

### 2.4. Sensitivity analyses

We explored the sensitivity of model results to data from different cities. Parameters were re-estimated after removing data one city at a time out of the cities with available seroprevalence data as well as Barranquilla, which was subjected to more intensive surveillance of ZIKV-associated NC compared to other cities [25]. Parameters were also re-estimated after removing all four cities with seroprevalence data from the model. Additional sensitivity analyses on the main model included using prior distributions for *p_S_* and *p_CZ_* that were informed from previous studies in the literature as well as partially pooling *p_Zl_* to reduce the uncertainty in the IAR estimates (electronic supplementary material).

Because the seroprevalence study only included participants between ages 2 and 45 years, we re-fitted the model separately for each age group (0–39 years and greater than or equal to 40 years). For the younger age group, the prior distributions for *p_Zl_* in Cúcuta, Medellín, Neiva and Sincelejo came from the post-epidemic seroprevalence as described in the electronic supplementary material, and Beta(1,1) prior distributions were used for the remaining cities. For the older age group, Beta(1,1) prior distributions for *p_Zl_* were used for all cities.

## 3. Results

**Figure 3.**
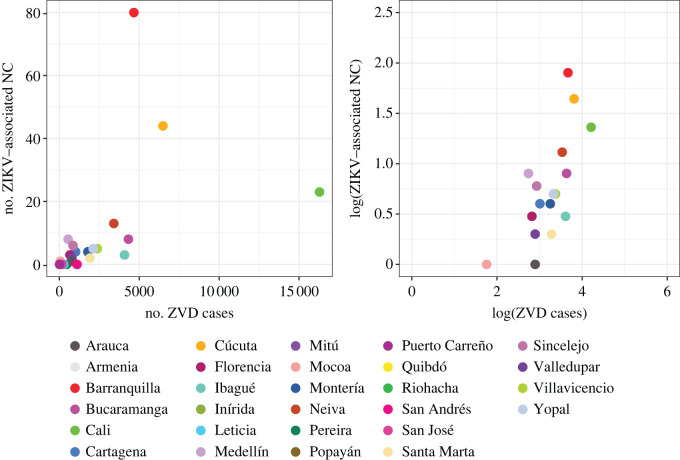
Number of reported cases of Zika virus (ZIKV)-associated neurological complications (NC) and Zika virus disease (ZVD) on a linear and a log–log scale for 28 capital cities. There is a positive statistically significant correlation of 0.51 (Pearson's correlation coefficient, 95% CI: 0.17–0.74, *p* = 0.006) on the linear scale and 0.70 (95% CI: 0.32–0.88, *p* = 0.002) on the log_10_ scale.

Eleven out of 28 capital cities reported zero cases of ZIKV-associated NC: Armenia, Inírida, Leticia, Mitú, Pereira, Popayán, Puerto Carreño, Quibdó, Riohacha, San Andrés and San José del Guaviare. Barranquilla reported the highest number of cases with 80. As expected, cities that reported more ZVD cases also tended to report more cases of ZIKV-associated NC (figure 3). Pearson's correlation coefficient for the relationship is 0.51 (95% CI: 0.17–0.74, *p* = 0.006).

**Figure 4.**
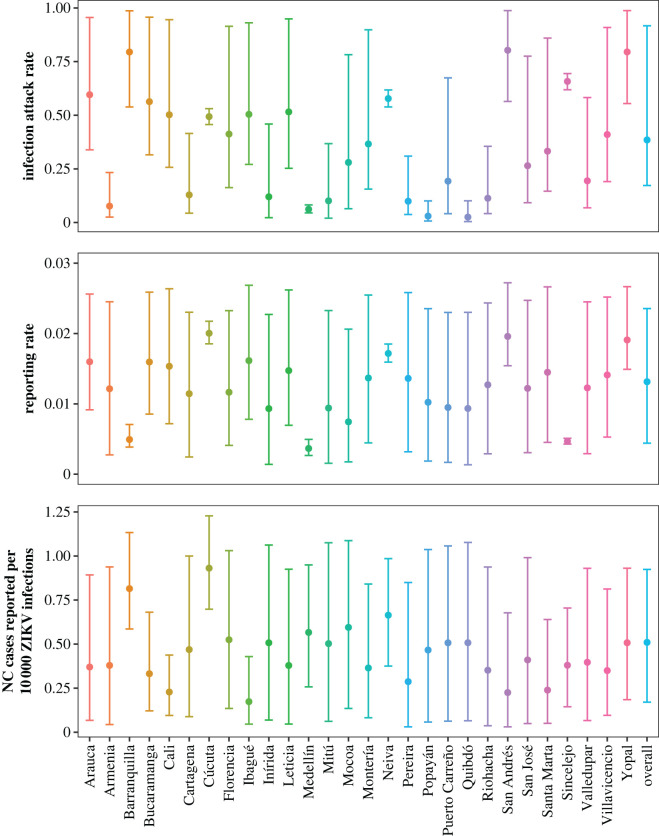
Estimated Zika virus (ZIKV) infection attack rates, Zika virus disease (ZVD) reporting rates and number of ZIKV-associated neurological complications (NC) cases reported per 10 000 ZIKV infections. Seroprevalence data were incorporated into the analysis for Cúcuta, Neiva, Medellín and Sincelejo. Posterior mean (points) and 95% credible interval (error bars) are shown for each city and overall.

The ZIKV IAR, ZVD reporting rate and reporting rate of ZIKV-associated NC were estimated for each city and overall (figure 4). There was substantial heterogeneity in estimates across cities, especially for IARs. The overall estimate for the ZIKV IAR had mean 0.38 (95% credible interval (CrI): 0.17–0.92). Both the ZVD reporting rate and the risk of reporting a case of ZIKV-associated NC per 10 000 ZIKV infections were low (mean 0.013, 95% CrI: 0.004–0.024 and mean 0.51, 95% CrI: 0.17–0.92, respectively) during the entire epidemic between 2015 and 2017. Overall, 54 (95% CrI: 5–210) cases of ZIKV-associated NC were expected to be reported for every 10 000 reported cases of ZVD on average (electronic supplementary material).

Across cities, mean estimates for the IAR ranged from 0.03 (95% CrI: 0.00–0.10) in Quibdó to 0.80 (95% CrI: 0.56–0.99) in San Andrés. Mean estimates for the reporting rate ranged from 0.004 (95% CrI: 0.003–0.005) in Medellín to 0.020 (95% CrI: 0.019–0.022) in Cúcuta. The mean estimated number of ZIKV-associated NC reported per 10 000 ZIKV infections was lowest in Ibagué (0.17, 95% CrI: 0.05–0.43) and highest in Cúcuta (0.93, 95% CrI: 0.70–1.23). Removing data from either Barranquilla or one of the four cities with seroprevalence data did not substantially impact parameter estimates. We also found that the model had enough power to estimate ZIKV IARs without serological data (electronic supplementary material).

When we assumed the same ZIKV IAR for males and females, females were more likely to be reported as ZVD cases compared to males (0.020, 95% CrI: 0.009–0.029 versus 0.011, 95% CrI: 0.005–0.016) but less likely to be reported as cases with NC (0.53 per 10 000 ZIKV infections, 95% CrI: 0.23–0.81 versus 0.69 per 10 000 ZIKV infections, 95% CrI: 0.30–1.01). Similarly, when we assumed the same ZIKV IAR for all ages, younger individuals were more likely to be reported as ZVD cases compared to older individuals (0.018, 95% CrI: 0.008–0.026 versus 0.012, 95% CrI: 0.005–0.017) but less likely to be reported as cases with NC (0.44 per 10 000 ZIKV infections, 95% CrI: 0.19–0.66 versus 0.93 per 10 000 ZIKV infections, 95% CrI: 0.40–1.39) (electronic supplementary material). For some cities, we also identified important differences in the posterior probabilities for ZVD reporting rates by sex and age group as well as the risk of ZIKV-associated NC by age group.

## 4. Discussion

Using surveillance data stratified by age and sex, seroprevalence data from four cities and a dataset on ZIKV-associated NC, we refined estimates of ZIKV IARs, ZVD reporting rates and the risk of ZIKV-associated NC for Colombia. We found that estimated ZIKV IARs were heterogeneous across cities, suggesting that some cities experienced worse epidemics than others, despite all being considered at-risk for arbovirus transmission. Cities with high estimated IARs previously may have populations with more natural immunity which would make them lower priority for vaccine trials; these cities may also experience large epidemics in the future after the susceptible population is replenished due to births, immigration and possible waning immunity [34,35]. While this study did not advance understanding of the mechanisms underlying biases in surveillance data (a community-based study such as that performed by Martínez Duran *et al*. [20] would be more suitable), it did attempt to overcome these biases by combining multiple datasets to estimate epidemiological parameters.

The overall estimate of the ZIKV IAR for the 28 capital cities analysed was 0.38 (95% CrI: 0.17–0.92), which is higher than that reported by both Mier-y-Teran-Romero *et al*. [21] (0.09, 95% CrI: 0.03–0.23) and Moore *et al*. (0.19, 95% CrI: 0.15–0.23) [22]. Moore *et al*. used a Beta(1,2) prior distribution to lightly constrain the IAR; however, their result from using a Beta(1,1) prior distribution was more similar (0.26, 95% CrI: 0.21–0.31) to the one presented here. Differences in the estimates could be attributed to the different spatial scales considered: while this study focused on capital cities, Moore *et al*. used department-level data and Mier-y-Teran-Romero *et al*. used national-level data. Moreover, all 28 cities in this study were at risk of arbovirus transmission and reported ZVD cases. By contrast, many locations which were included in the other two studies were not at risk and did not report ZVD cases, including Bogotá, with a population of about eight million. Higher overall IARs would be expected from an analysis that only included at-risk locations.

The 95% CrIs for the overall estimate of the IAR were wider compared to those reported by both Mier-y-Teran-Romero *et al*. and Moore *et al*. and likely reflect heterogeneity in IARs across cities. While the post-epidemic seroprevalence for Medellín was about 0.07, the estimates for the other three cities ranged from 0.48 to 0.66. There is evidence that high IARs resulting in herd immunity brought an end to the ZIKV epidemic in the Americas [23]. Seroprevalence studies conducted in other large cities in Latin America have also reported high IARs such as 0.46 (95% CI: 0.44–0.48) in Managua, Nicaragua [36] and 0.73 (95% CI: 0.70–0.76) in Salvador, Brazil [37]. However, IARs were not uniform even at small spatial scales, leaving pockets of susceptible populations [37,38]. This heterogeneity may not have been captured as well by Mier-y-Teran-Romero *et al*. and Moore *et al*., who used coarser surveillance data and did not incorporate seroprevalence data from Colombia.

The estimated reporting rate of ZVD across cities was 0.013 (95% CrI: 0.004–0.024) in this study. While Mier-y-Teran-Romero and Moore *et al*. both estimated larger reporting rates, all estimates were consistent when considering their uncertainty. Mier-y-Teran-Romero *et al*.'s [21] estimate was over two times larger (0.03, 95% CrI: 0.01–0.07). Moore *et al*.'s [22] estimate of the probability that a symptomatic ZIKV infection is reported as a suspected or laboratory-confirmed ZVD case had mean 0.040 (95% CrI: 0.019–0.077) (obtained from the model's posterior samples). Our use of seroprevalence data to estimate IARs could explain the lower uncertainty in estimated reporting rate of ZVD here compared to the other studies. More precise estimates of reporting rates would be expected from better estimates of the IARs. Our estimate is also similar to one reported by O'Reilly *et al*. [23]. They estimated a median reporting rate of 0.017 (95% CrI: 0.013–0.025) for Colombia using a spatio-temporal dynamic transmission model for ZIKV infection in 90 cities within 35 Latin American and Caribbean countries [23]. The overall estimate of the number of ZIKV-associated NC reported per 10 000 ZIKV infections was 0.51 (95% CrI: 0.17–0.92). This estimate is lower than that reported by Mier-y-Teran-Romero *et al*. [21] (2.0, 95% CrI: 0.6–4.6) and Moore *et al*. [22] (2.9 GBS cases per 10 000 symptomatic ZIKV infections, 95% CrI: 1.4–5.5, obtained from the posterior samples) for Colombia. One possible reason for the discrepancy is that three different sources for ZIKV-associated NC were used by the studies. Mier-y-Teran-Romero *et al*. used a total of 677 cumulative cases, which were reported in the INS Weekly Epidemiological Bulletin at the end of 2016, and Moore *et al*. used 773 cases which were reported to PAHO. Although these were the only case numbers available at the time, they were nevertheless subject to misclassification. Here, 418 cases remained following a verification process. Another contributing factor is that we estimated higher IARs compared to Mier-y-Teran-Romero *et al*. and Moore *et al*. Higher estimated IARs would lead to lower estimates of the risk of NC following ZIKV infection.

We estimated that there were 54 (95% CrI: 5–210) reported cases of ZIKV-associated NC for every 10 000 reported cases of ZVD on average. This estimate is less than half that from Mier-y-Teran-Romero *et al*.'s study (111, 95% CrI: 0–567), but the large uncertainty associated with those estimates make them broadly consistent. Their estimate incorporated baseline levels of GBS for all locations except Colombia and Puerto Rico [21]. Thus, the interpretation of their estimate more closely aligns with the total expected number of reported GBS cases from all causes for every 10 000 reported ZVD cases, while the estimate here can be interpreted as excess cases of NC due to ZIKV per 10 000 reported ZVD cases.

Interestingly, the point estimates from the raw data (calculated as reported cases of ZIKV-associated NC divided by ZVD cases) for 12 out of 28 cities in this study fell outside of their 95% CrI. Eleven of those cities reported zero cases of ZIKV-associated NC but based on the number of reported ZVD cases in those cities, the model predicted there would be reported ZVD cases with NC.

It is possible that ZVD cases with NC were not reported because they did not occur due to chance. Thirteen out of 17 cities that did report ZVD cases with NC reported fewer than 10, making it a rare event. Another possible explanation is that severe cases of ZVD were under ascertained because of barriers to healthcare access, particularly in rural areas where neurologists are scarce, i.e. in Quibdó and Chocó [39]. A study on the availability and distribution of medical specialists offering high- and medium-complexity services in Colombia estimated that the country had only one neurologist and one neurosurgeon for every 100 000 people in 2011 [39]. This estimate is lower than the global median of the total neurological workforce (comprising the total number of adult neurologists, neurosurgeons and paediatric neurologists), which was estimated by the World Health Organization (WHO) at 3.1 per 100 000 population from data collected between 2014 and 2015 [40]. In Colombia, medical specialists tend to be concentrated in capital cities, but even the capitals of rural departments struggle with lack of specialists. According to one hospital manager in Leticia, the capital of the Amazonas department, a major limitation faced by specialists is work-related travel. For every three weeks that a specialist works, another specialist must agree to replace them for one week while they rest [39].

Our findings that females were more likely than males to be reported as ZVD cases to the surveillance system but less likely to be reported as cases with NC are consistent with our previous work [41]. The same trend in reporting rates was found for most cities, but no important differences in the risk of NC by sex were found at the city level (possibly due to lack of power). Overall, younger individuals had higher ZVD reporting rates than older individuals, assuming the same IARs, while older individuals were more likely to be reported as cases with NC. These findings were also expected based on our previous work [41] and GBS epidemiology. We obtained similar results at the city level for reporting rates by age, but few cities had important differences in the risk of NC, which, again, could be related to small sample sizes and lack of power. The cities with important differences in estimates by sex and age group tended to include those that had more available data.

ZVD is not the only disease for which multiple data types have been employed to overcome biases or gaps in surveillance data. Watson *et al*. [42] used community-uploaded obituary certificates to validate a mathematical model of SARS-CoV-2 transmission dynamics in Damascus, Syria. The model, which was fitted to reported COVID-19 deaths, estimated that only 1.25% of deaths (sensitivity range 1–3%) from COVID-19 were reported in Damascus between July 2020 and August 2020. The alternative data source confirmed substantial under-ascertainment of mortality over that time period [42]. Another example comes from the Global Polio Eradication Initiative, which began in 1988 after the WHO declared poliovirus a target for eradication [43]. Surveillance of acute flaccid paralysis (AFP) is the primary means by which poliovirus is monitored globally. However, as only one in 200–1000 individuals infected by poliovirus becomes paralyzed, most infections are not detected by AFP surveillance. Consequently, both environmental sampling of sewage and genetic sequencing of polioviruses have been employed to improve the identification of poliovirus outbreaks, understanding of their spread and determination of the appropriate vaccination response [43]. The reporting rate of poliomyelitis has also been estimated by incorporating seroprevalence data and outbreak data into a mathematical model [44].

A limitation of this analysis is that seroprevalence data were only available for four cities. Parameter estimates, especially the IARs, for cities without these data were much more uncertain. Although removing data from the four cities did not seem to greatly affect parameter estimates for the remaining cities, the estimated IARs for some cities were nonetheless surprising. For example, Barranquilla and Cartagena are located just 100 km apart along Colombia's Caribbean coast and have similar altitudes. Yet, the estimated IAR for Barranquilla was much higher than Cartagena (0.79, 95% CrI: 0.54–0.99 versus 0.13, 95% CrI: 0.04–0.42). Estimated IARs for Pereira and Riohacha were also lower than expected, given that both cities are considered hyperendemic for another mosquito-borne viral disease, dengue [45]. Seroprevalence data from these cities would help improve our estimates.

Another limitation is that the analysis included ZVD cases from 2015 to 2017, but the seroprevalence study was conducted in late 2016. However, the seroprevalence data should not bias the results because ZIKV transmission was limited in 2017. On average, 1.3% (range: 0.09–4.0%) of ZVD cases across the four cities in the seroprevalence study were reported from the beginning of 2017 up until mid-2017 (the endpoint of our data).

All available data from Colombia, including an additional 50 415 cases of ZVD and 194 cases of ZIKV-associated NC, were not used in this analysis due to restricting to at-risk capital cities. Also, as reporting rates are expected to be higher in capital cities compared to non-capital cities and departments, the findings here may not be generalizable to the rest of the country. A strength of this analysis is the fine spatial scale of the data and the fact that the NC dataset was checked against standardized case definitions. Future research should analyse ZIKV surveillance biases in other countries; however, comparable datasets may not be available. Some countries such as Ecuador reported very few cases of ZIKV-associated NC to PAHO [46].

In conclusion, our study highlights the need for additional data sources to overcome biases in surveillance data. We observed differences in ZIKV IARs and reporting rates of ZVD across capital cities as well as sex and age group. We also estimated the risk of ZIKV-associated NC, which may have been under-ascertained in rural cities where greater incentives are needed to attract and retain medical specialists.

## Data Availability

Stan code and aggregated data can be found on GitHub: https://github.com/kcharniga/zika_capital_cities. ZVD surveillance data are available on GitHub: https://github.com/kcharniga/descriptive_zika. These data include the number of weekly reported cases by administrative level 2 (city) as well as sex and age category. The NC dataset cannot be shared publicly as the data contain information on a small number of patients. Although the data are anonymized, identification is a risk given the high geographic resolution and large combination of predictor variables. This determination was made by Comité de Ética y Metodologías de Investigación. To request access to these data, please contact: secretariactin-cein@ins.gov.co, (57+1) 2207700 Ext. 1331-1108, Colombia. Supplementary material is available online [47].
